# 95 Impact of Neighborhood Antibiotic Resistance on Individual Risk: A Geospatial Analysis of E. coli

**DOI:** 10.1017/ash.2026.10517

**Published:** 2026-06-23

**Authors:** Elizabeth Monsees, Sara Reese, Lisa Hall, Annie Wirtz, Monika Pogorzelska-Maziarz

**Affiliations:** 1 Children’s Mercy Hospital; 2 APIC; 3 The University of Queensland; 4 Children’s Mercy Kansas City

## Abstract

**Background:** Almost a decade has passed since the inaugural Core Elements of Outpatient Antibiotic Stewardship from the Centers for Disease Control and Prevention. Recognizing that outpatient prescribing accounts for upwards to 90% of antibiotic use, with 30% of those prescriptions deemed inappropriate, four core elements (commitment, action, tracking, education) guide the identification and monitoring of stewardship activities. We aimed to examine infection preventionist (IP) perceptions of outpatient stewardship programs. **Methods:** Members and non-members of the Association for Professionals in Infection Prevention (APIC) were invited to participate in the quinquennial electronic survey of IPs between June 6 and July 31, 2025, through email, QR codes, and social media campaigns. Data were collected on nine outpatient antimicrobial stewardship program (ASP) characteristics, including structure, leadership, and educational resources. Descriptive statistics were used to summarize the data. **Result:** Responses were received from 199 IPs who selected outpatient settings as their primary work location. Among these, 58% (n=115) reported the presence of an ASP, while 10% (n=19) were unsure. IPs reported that most ASPs were led by either clinical pharmacists (38.5%, n=37), infectious disease physicians (24%, n=23), or infectious disease pharmacists (19%, n=18), although 13% of IPs (n=15) could not identify the program leader. Nearly one-quarter of respondents (27%) indicated that they had access to an infectious diseases pharmacist. Over 75% (79%, n=86) reported the use of up-to-date treatment recommendations for infections; however, 24.8% (n=27) were unsure whether education on appropriate antibiotic prescribing was offered. Participation in multidisciplinary ASP rounding was evenly divided: yes (31%, n=34); no (35.8%, n=39); and do not know (33%, n=36). Over a third (35.3%) of IPs attended rounds. **Conclusion:** Although most outpatient stewardship programs are led by frontline clinical pharmacists, this survey found they were frequently supported by infectious diseases experts. This likely reflects the perception that these programs operate in hospital systems where IPs are present. Surprisingly, multidisciplinary rounding was reported even in outpatient environments, challenging assumptions that such practices are limited to inpatient stewardship. While education is a core component of stewardship, multidisciplinary educational efforts are necessary to make resources more visible and accessible across disciplines.

